# Sylvian fissure subpial hematoma: a rare imaging presentation of a ruptured middle cerebral artery aneurysm

**DOI:** 10.1055/s-0044-1788268

**Published:** 2024-08-31

**Authors:** Jacob A. Schroeder, Thomas P. Reith, Matthew D. Benson, Joan E. Maley, Leonardo Furtado Freitas

**Affiliations:** 1 University of Iowa Hospitals and Clinics, Department of Radiology, Division of Neuroradiology, Iowa City IA, United States.

**Figure 1:**
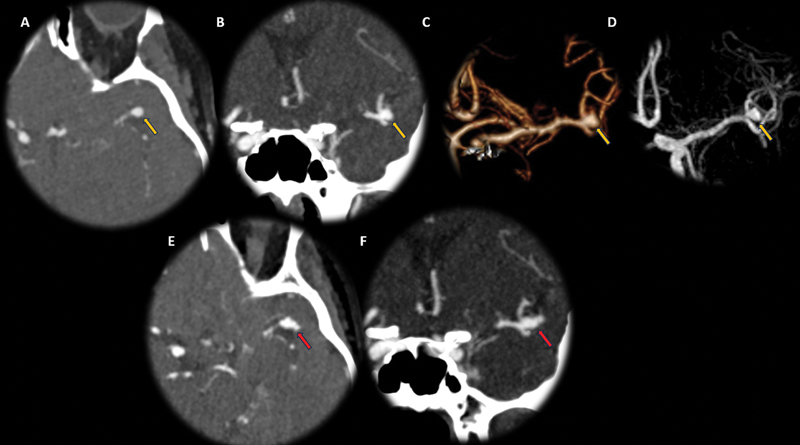
Previous head computed tomography angiography (CTA) (A–C) and head magnetic resonance angiography (MRA) (D); head CTA (E-F) follow-up on acute presentation. Increased size and new lobulation of the saccular left middle cerebral artery (MCA) aneurysm bifurcation (orange and red arrows).

**Figure 2:**
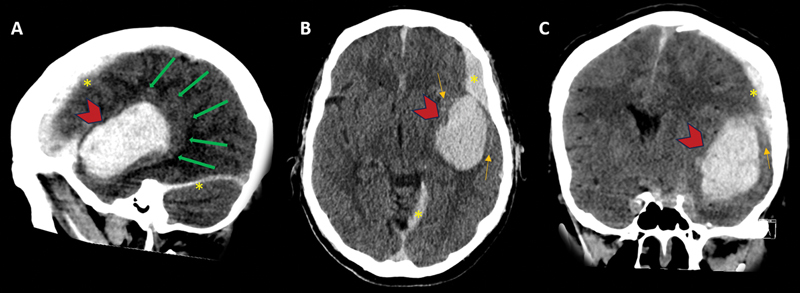
Head CT without IV contrast immediately after patient's severe headache. Interval large acute hematoma along the left sylvian fissure, probably in the subpial compartment (red arrowheads). There was local mass effect with adjacent opercular cortex compression (green arrows). Small component of subdural (yellow asterisks) and subarachnoid (orange arrows) hemorrhages were also visualized.

A 68-year-old female patient with a previously unruptured aneurysm in the left middle cerebral artery ( Figure 1 ) presented with aphasia and severe headache. A computed tomography angiography (CTA) showed growth and new lobulation ( Figure 1 ) associated with hemorrhages, including a large sylvian hematoma, probably in the subpial compartment ( Figure 2 ). The findings suggested aneurysm rupture.

**Figure 3:**
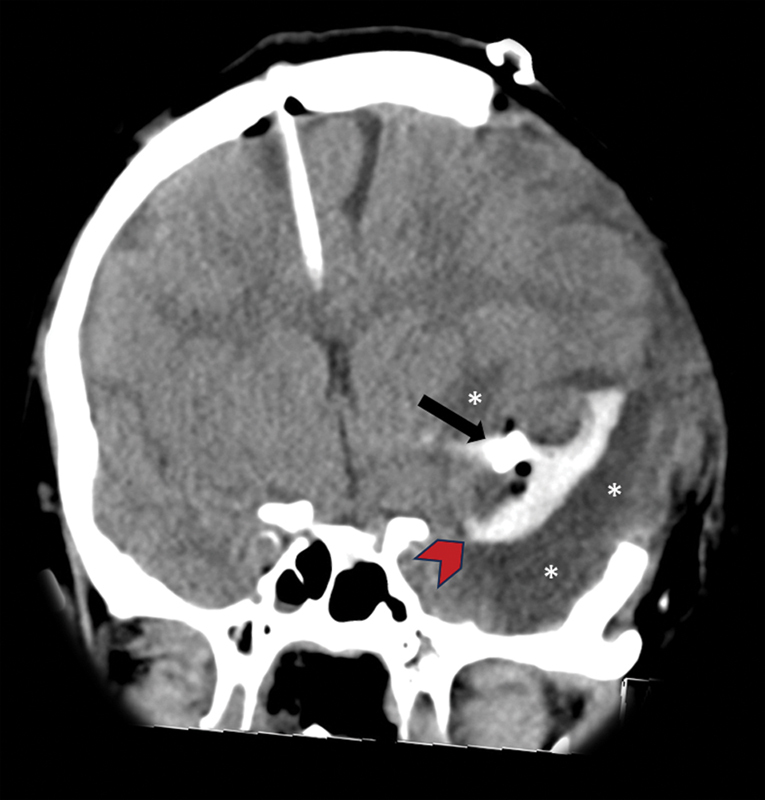
Immediate post operative CT head showing left decompressive craniectomy, right frontal ventriculostomy, aneurysm clipping (black arrow), and partial left sylvian fissure subpial hematoma evacuation (red arrowhead). Note is made for significant edematous changes along the left temporal and insular lobes (white asterisks).

Subpial hemorrhages happen within the potential space between the pia mater and cortex and are extremely rare in adults. 1 It is hypothesized that the presence of blood below the pia may damage thin arteries, with secondary bleeding and hemorrhage expansion. 2 They may cause injury to the cortex and severe edema and vasospasm, resulting in a poor prognosis ( Figure 3 ). 1 2 3
